# Design of a 1-bit MEMS Gyroscope using the Model Predictive Control Approach

**DOI:** 10.3390/s19030730

**Published:** 2019-02-11

**Authors:** Xiaofeng Wu, Zhicheng Xie, Xueliang Bai, Trevor Kwan

**Affiliations:** 1 Intelligent Space Systems Laboratory, School of Aerospace, Mechanical and Mechatronic Engineering, University of Sydney, Sydney, NSW 2006, Australia; zxie9629@uni.sydney.edu.au; 2 Centre for Quantum Technologies, National University of Singapore, Singapore 117543, Singapore; cqtbx@nus.edu.sg; 3 School of Engineering, Sun Yat-sen University, Guangzhou 510275, China; kwanth@mail.sysu.edu.cn

**Keywords:** MEMS gyroscope, digital control, model predictive control, sigma delta modulation

## Abstract

In this paper, a bi-level Delta-Sigma modulator-based MEMS gyroscope design is presented based on a Model Predictive Control (MPC) approach. The MPC is popular because of its capability of handling hard constraints. In this work, we propose to combine the 1-bit nature of the bi-level Delta-Sigma modulator output with the MPC to develop a 1-bit processing-based MPC (OBMPC). This paper will focus on the affine relationship between the 1-bit feedback and the in-loop MPC controller, as this can potentially remove the multipliers from the controller. In doing so, the computational requirement of the MPC control is significantly alleviated, which makes the 1-bit MEMS Gyroscope feasible for implementation. In addition, a stable constrained MPC is designed, so that the input will not overload the quantizer while maintaining a higher Signal-to-Noise Ratio (SNR).

## 1. Introduction

A high-performance micro-machined MEMS gyroscope is appealing to many researchers as it is advantageous in terms of power, cost and flexibility over the bulky and expensive macroscopic gyroscopes. When an MEMS gyroscope is moving in a direction and an angular velocity is applied to the gyroscope, the sensing element will experience a displacement as a result of Coriolis force. The incorporation of the Delta-Sigma (Δ∑) modulator to the gyroscope sensing element is one of the most promising approaches to implement the MEMS gyroscope due to the circuit simplicity and the benefits of incorporating the sensing element in a feedback control loop [1] to improve the sensing stability. The Δ∑ modulation-embedded MEMS gyroscope was first introduced in [2], and ever since has become a popular research topic in the literature [3,4,5,6,7]. 

As an efficient A/D conversion method, the Δ∑ modulator can be embedded into many industrial applications, e.g., [8,9,10,11]. Along with the development of the Microelectromechanical Systems (MEMS), the Δ∑ modulator-based gyroscopes and accelerometers may be used in many applications. For instance, it is possible to choose MEMS gyroscopes as the sensing device for small satellite missions, e.g., [12]. However, due to the non-linear nature of the quantizer, the extra integrators in the gyroscope transfer function may cause stability issues in the control loop. Moreover, the compensator will also introduce extra poles in the control loop and consequently affect the noise-shaping performance of the system [5]. Additional integrators, serving as usual noise shaping solutions, are adopted in the feedback loop to attenuate the magnitude of the impulse response of the noise transfer function (NTF) at low frequencies (see e.g., [13] for different Δ∑ modulator-based MEMS gyroscope structures). This methodology is analogous to a PID (Proportional-Integral-Derivative) control system, in which the performance of the designed system depends on the experience of the designer [14].

Among various ∆∑ modulation methods, the ones with bi-level quantizers are more attractive because of their circuit simplicity and the binary nature of the quantizer outputs. They have been proven to be an efficient alternative to the multi-level quantizers [15]. Such bi-level quantizers are used to develop the multiplier free analog to digital (A/D) converters, e.g., [16], to achieve the simplest digital hardware circuitry. Based on the bi-level quantizer, the concept of 1-bit processing has been widely investigated in the context of finite-impulse-response (FIR) filters [17], infinite-impulse-response (IIR) filters e.g., [18], and digital communication e.g., [19,20]. One of the many successful applications of 1-bit processing is in audio applications (e.g., [21,22]), where the 1-bit coding scheme is used to develop high frequency (64 or 128 times 44.1 kHz) encoding technology for the audio industry. A multiplier free control system has been proposed by [23], namely, the 1-bit processing control system. As the signals are in the 1-bit format, multipliers can be removed by choosing a modified controller structure [24].A 1-bit processing-based MPC (OBMPC) method was then developed in [25] to extend the 1-bit processing-based control system to advanced control algorithm applications. 

Inspired by the 1-bit processing-based A/D conversion and 1-bit processing-based control systems, an implementation of a novel OBMPC for the MEMS gyroscope is developed in this work. The authors provide the design of an MPC algorithm for the bi-level ∆∑ modulator-based MEMS gyroscope (1-bit MEMS gyroscope) and discusses the potential issues of implementing such an OBMPC-based MEMS gyroscope. A new OBMPC structure is designed to implement the 1-bit MEMS gyroscope with a high sampling rate. As one of major benefits of the MPC algorithm, the proposed OBMPC structure can include the constraints in the quantizer inputs, which serve as the stabilization technique for the Δ∑ modulator while providing a better SNR when quantizer overloading occurs. 

The rest of this paper is organized as follows. Section 2 describes the Δ∑ Modulator-based MEMS gyroscope. Section 3 details the design of the OBMPC-based MEMS gyroscope. Experimental results are described in Section 4. Finally, conclusions are presented in Section 5.

## 2. Δ∑ Modulator-Based MEMS Gyroscope

**Figure 1:**
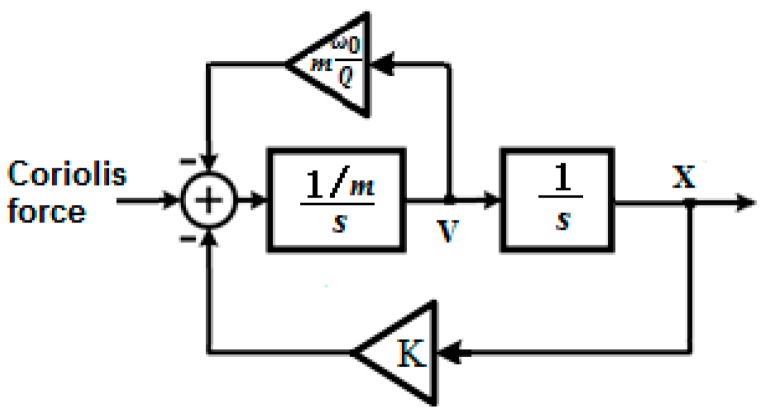
System level diagram of the dynamic system of a mechanical sensor.

A typical approach to design a Δ∑ modulator-based MEMS gyroscope is to treat the MEMS gyroscope as a Δ∑ modulation-based control loop. For most MEMS gyroscopes, the angular motion is determined by measuring the vibration of the proof mass, which is excited due to Coriolis force. The sense mode of the MEMS gyroscope can then be regarded as a spring damper dynamic system responding to Coriolis force, and hence can be modeled by two integrators in series. Figure 1 shows a system level diagram of a mechanical sensor.

Where K is the spring stiffness, and $\frac{\omega_{0}}{2\pi }$ is the resonant frequency of the dynamic system. For the sensing mode of the gyroscope, the control loop design problem can also be treated as the Δ∑ modulator-based accelerometer under Coriolis force, e.g., [26,27]. The continuous-time transfer function of the mechanical sensor can be denoted as:

(1) $$H_{m}\left(s\right)=\frac{\frac{1}{m}}{s^{2}+\frac{\omega_{0}}{Q}s+\omega_{0}{}^{2}}$$

where *m* is the mass of the sensing element, $\omega_{0}$ is the resonant frequency and Q is the quality factor. High-quality factors are generally required to achieve high sensitivity of the sensor (200–250 for the sense mode and 35,000–45,000 for the drive mode [28]). Due to the phase shift introduced by the mechanical sensing element, a simple lead compensator must be included to stabilize the control loop. Other sensor fusion technologies are also available, e.g., [29,30,31], but are outside of the scope of this work. The output of the compensator can be regarded as the input of the Δ∑ modulator, which serves as an interface to digitalize the sensor signal. The resulting digital bit stream can be translated into an electrostatic force as the feedback to the control loop of the sensor [32]. 

**Figure 2:**
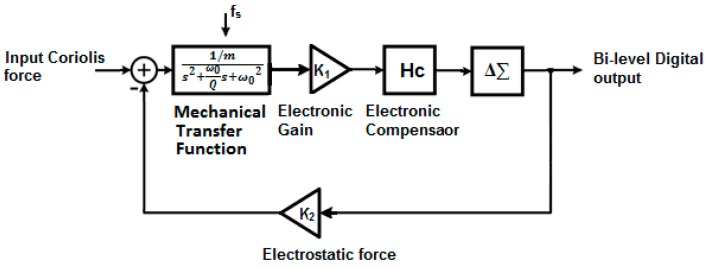
Structure of a typical Δ∑ modulator-based MEMS gyroscope.

To further analyze the stability and performance of the Δ∑ modulator-based MEMS gyroscope, one can treat the sensing component and the compensator as two second order loop filters, and then analyze the entire control loop as a high order Δ∑ modulator. The structure of the Δ∑ modulator-based MEMS gyroscope is shown in Figure 2.

Like any other A/D conversion method, the Δ∑ Modulation process introduces quantization noise into the MEMS control loop. Such quantization noise, coupled with mechanical noise and electrical noise, may cause large gyroscope bias and instability of the control loop. Filtering techniques are therefore required to decrease the in band noise. Filters can be embedded into the control loop before the A/D conversion as the Δ∑ modulator operates at over sampling rate (OSR) and can be constrained into a narrow frequency band. Also, the bandwidth requirements of the electronic components needed for implementation in the integrated circuit are relatively demanding as a high OSR is eventually required to achieve a good SNR.

**Figure 3:**
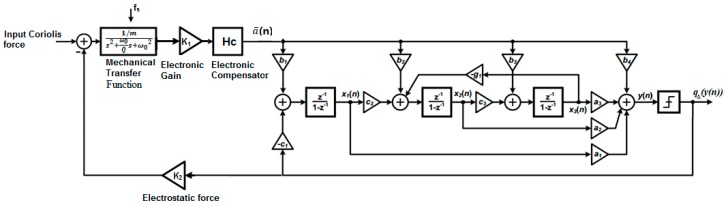
A third order 1-bit MEMS gyroscope with resonator.

One way to minimize the quantization noise is to increase the order of the Δ∑ modulator in the control loop at a cost of circuit complexity. A well designed high-order Δ∑ modulator-based MEMS gyroscope can filter most of the noise from the Δ∑ modulator loop. For instance, the results obtained in [33] proved that a SNR of 93 dB can be achieved with a relatively low OSR of 500, including realistic values for electronic noise introduced. Also, the integrators can be replaced with resonators, e.g., [26,28], to build a band-pass Δ∑ modulator. In this work, a bi-level Δ∑ modulator is used in the 1-bit MEMS gyroscope. A third order 1-bit MEMS gyroscope with an embedded resonator is shown in Figure 3.

More Δ∑ modulator-based MEMS gyroscope implementations can be found in a review paper [1]. 

## 3. OBMPC Structure for the 1-bit MEMS Gyroscope

In order to design a Δ∑ modulator-based MEMS gyroscope with guaranteed stability while maintaining a reasonable SNR, in this section, we adopt an MPC approach to implement the MEMS gyroscope. This approach uses the 1-bit feedback from the Δ∑ modulator and therefore can be defined as the 1-bit processing-based Model Predictive Control (OBMPC) in this paper. 

### 3.1. Δ∑ Modulator with Parallel State Variables

**Figure 4:**
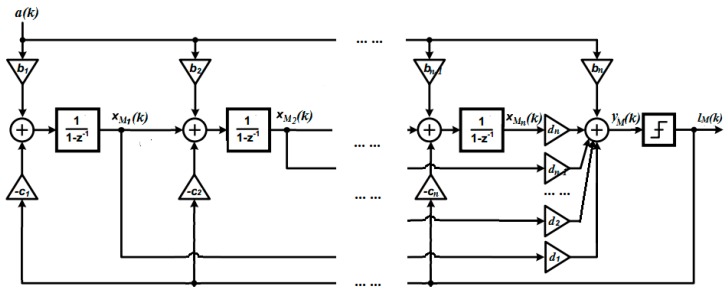
Structure of an *n*th order 1-bit ∆∑ modulator.

The control objective is to find the integrator output which has minimized quantization error. The digital signal (e.g., a sampled continuous-time signal) a(k) shall be considered as the output of the MEMS gyroscope system and the input to a high order Δ∑ modulator. An *n*th order Δ∑ modulator structure is shown in Figure 4.

The state-space equations of the proposed structure can be written as

(2) $$\{\begin{array}{c} x_{M}\left(k+1\right)=A_{M}x_{M}\left(k\right)+B_{M}a\left(k\right)-C_{M}l_{m}\left(k\right)\\ y_{M}\left(k\right)=D_{M}\left[\begin{array}{c} x_{M}{}_{1}\\ x_{M}{}_{2}\\ \vdots \\ x_{M}{}_{n} \end{array}\right] \end{array}$$

where $x_{M}$(*k*) ∈ $R^{n}$ is the state vector, $A_{M}=\left[\begin{array}{ccccc} 1 & 0 & 0 & \ldots & 0\\ 1 & 1 & 0 & \ldots & 0\\ 0 & 1 & 1 & \ldots & 0\\ \vdots & \ddots & \ddots & \ddots & \vdots \\ 0 & \ldots & 0 & 1 & 0 \end{array}\right]$, $B_{M}=\left[\begin{array}{c} b_{1}\\ b_{2}\\ b_{3}\\ \vdots \\ b_{n} \end{array}\right]$, $C_{M}=\left[\begin{array}{c} c_{1}\\ c_{2}\\ c_{3}\\ \vdots \\ c_{n} \end{array}\right],D_{M}=\left[d_{1},d_{2},\ldots d_{n}\right],x_{M}\in R,y_{M}\left(k\right)\in R,\;k\in N$. $l_{M}\left(k\right),\;\mathrm{where}\;l_{M}\left(k\right)≜q_{\Delta }\left(y_{M}\left(k\right)\right),$ is the quantizer output and a(k)∈R is the modulator input. To ensure that the input does not overload the controller, the state variables are required to be clipped. Specifically speaking, as presented in [34], for a second-order Δ∑ Modulator, with unit feedback gain, the state bound can be presented as

(3) $$\{\begin{array}{c} {\left|x_{1}\right|}_{max}=\left|u_{in}\right|+2\\ {\left|x_{2}\right|}_{max}=\frac{{\left(5-\left|u_{in}\;\right|\right)}^{2}}{8\left(1-\left|u_{in}\;\right|\right)} \end{array}$$

where $u_{in}$ is the modulator input, $x_{1}$ and $x_{2}$ are, respectively, the states for the first and second integrators. The clipping principle is further generalized in [35] with different feedback gains. Ref. [36] provided state bounds analysis for the third-order Δ∑ Modulators. Although the principles discussed above are designed for sinusoidal inputs, they are fairly strict from the design point of view and can be considered as sufficient conditions for most designs [37]. To further simplify the problem, as suggested in [37,38], the clipping threshold can only be set to the last integrator. The variable gain method [39] can be used as the design guide of the hard constraints of the last integrator in the control loop. In practical cases, one can find out a “safe” threshold by studying the impulse response of a stable modulator.

The asymptotical stability of such Δ∑ modulators can be designed by moving all the eigenvalues of $A_{M}$ inside the unit circle. The modulator is oversampled so that a(k) can be considered as constant within *n* time steps where n≪OSR, i.e., a(k)=a(k+1)…=a(k+n). *x_1_(k), x_2_(k)…x_n_(k)* are the state variables for the *n*th integrator. $l_{M}$ is weighted by a bi-level quantizer, where $l_{M}\in \left\{\Delta ,-\Delta \right\}$. The quantization level Δ is standardized as 1 and ${\vec{l}}_{M}\left(k\right)$ is scaled by $c_{1},c_{2},\ldots c_{n}$ according to the quantization level. In this particular case, the state matrix ${\vec{A}}_{M}$ can be transformed into the Jordan canonical form by replacing ${\vec{x}}_{M}$ with $P_{T}{}^{-1}x_{M}$ as used in [39],where $P_{T}=\left(\begin{array}{cccc} \vdots & \vdots & \vdots & \vdots \\ \rho_{1} & \rho_{2} & \cdots & \rho_{n}\\ \vdots & \vdots & \vdots & \vdots \end{array}\right)$ and $\rho_{1}$, $\rho_{2}$…$\rho_{n}$ are the eigenvalues of $A_{M}$. Equation (3) then becomes:

(4) $$\{\begin{array}{c} x_{M}\left(k+1\right)=A_{M}x_{M}\left(k\right)+B_{M}a\left(k\right)-C_{M}l_{M}\left(k\right)\\ y_{M}\left(k\right)=D_{M}x_{M}\left(k\right) \end{array}$$

where $A_{M}=P_{T}A_{M}P_{T}^{-1}$, $B_{M}=P_{T}B_{M}$, $C_{M}=P_{T}C_{M}$. $x_{M}\in R,y_{M}\left(k\right)\in R$.

**Figure 5:**
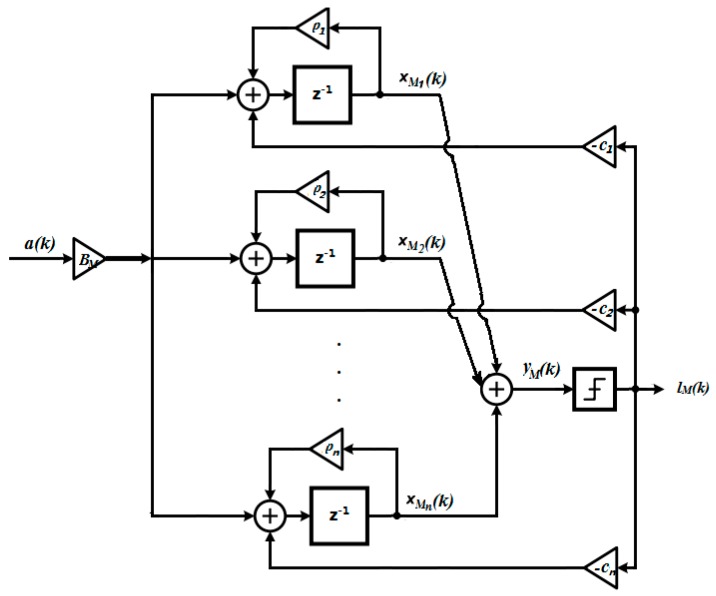
N*th* order Δ∑ modulator with parallel state variables (Thick lines denote vector routing).

The main benefit for this structure is that the state variables are now decoupled. To further simplify the problem, define $D_{M}≜\left[1,1,\cdots ,1\right]$. The structure of the Δ∑ modulator with parallel state variables can then be reconstructed as shown in Figure 5.

In Figure 5, $\rho_{1}$, $\rho_{2}$…$\rho_{n}$ are the eigenvalues of $A_{M}$. It is worth noting that the inclusion of linear feedback paths other than the resonators results in a diagonal canonical form of $A_{M}$. It is possible to extend such parallel structures to many Δ∑ modulator structures if the state matrix $A_{M}$ is similar to a diagonal matrix. In the following subsection, we shall assume that a simple diagonal state matrix is used. Then, each state variable can be easily clipped, or constrained, by the designer. Designing and implementing a clipper is one of the easiest ways to stabilize a Δ∑ modulator. The main challenge, however, lies in how to choose a reasonable clipping level while retaining the high SNR when the input does not overload the quantizer. In practical missions, if the non-ideal integrators and noises are taken into consideration, rigorous clipper level (typically much higher than the input signal for higher order Δ∑ Modulators, e.g., 90 times of the input signal for a third order Δ∑ Modulator), may result in low SNR at the noisy instants, even when the stability of the control loop can be guaranteed. Moreover, as a non-linear approach, the clipping technique will bring additional non-linearity to the Δ∑ Modulator, which is also a non-linear system itself, so that the stability analysis is even harder to perform (e.g., [40]). Hence, an OBMPC controller is proposed to be applied to such Δ∑ modulators. The proposed controller has the ability to handle hard constraints (i.e., the clipping thresholds) on all the state variables. Also, the order reduction is discussed in [39] for some particular Δ∑ modulators, which may help to decrease the online computation effort (if required) of the proposed method.

In some other cases, however, the use of resonators will result in a non-diagonal $A_{M}$. These cases are studied in [39] and can be analyzed on a system-by-system basis. Generally, not all the state variables can be decoupled for the Δ∑ modulator structure with resonators. On the other hand, they can be written as a parallel state structure with a certain level of decoupling. Hence, it is still possible to decouple some of the state variables in most non-diagonal structures. For example, consider a third-order Δ∑ modulator with a resonator on the second and the third integrator. In this case, only the first integrator can be decoupled, which means it can be restructured to be in parallel with the other two integrators. In such a structure, the first and the third state variables are explicitly known. However, this will only require the study of the impulse response of a first-order NTF and a second-order NTF, respectively, which is easier than studying the third-order NTF directly to determine a reasonable a clipper level. The worst case is that none of the state variables can be decoupled. Hence, only the last state variable can be directly constrained. It is still possible to stabilize the modulator as suggested in [38]. However, the clipping action will result in a relatively low SNR in comparison to the individually clipped Δ∑ modulator. In the following section, we assume that the state matrix of the Δ∑ modulator in the proposed mission is designed to be diagonal. This was chosen to simplify the analysis such that case-by-case studies need not be performed for the proposed method. 

### 3.2. OBMPC-based MEMS Gyroscope Using 1-bit Processing

#### 3.2.1. Linearization Assumption and Problem Formulation

**Figure 6:**
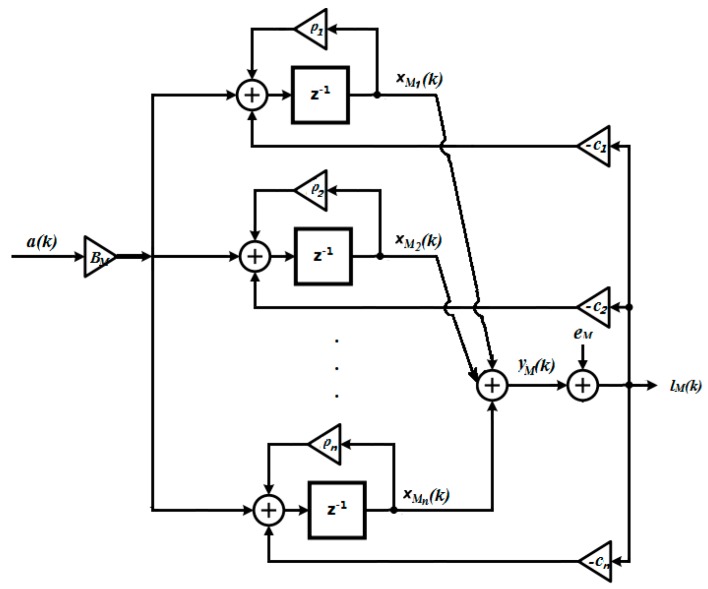
Linearized *n*th order Δ∑ modulator with parallel state.

As discussed above, if the state variables can be decoupled, such that the constraints applied on the state variable are linearly independent, the OBMPC can be implemented with a relatively simple circuit under the framework of 1-bit processing control system. This means that the Karush-Kuhn-Tucker (KKT) [41] condition is sufficient to address such a problem. By linearizing the quantization noise [30], Figure 5 can be presented as seen in Figure 6.

In Figure 6, $e_{M}\left(k\right)$ is the filtered quantization noise. According to Figure 6,

(5) $$l_{M}\left(k\right)=y_{M}\left(k\right)+e_{M}\left(k\right).$$

Define N(z) and S(z) as discrete linear time invariant filter representing the respective NTF and the Signal transfer Function (STF). *S(z)* can be presented in state space form as

(6) $$\{\begin{array}{c} x_{M}\left(k+1\right)=A_{P}x_{M}\left(k\right)+B_{S}a_{M}\left(k\right)\\ y_{M}\left(k\right)=C_{S}x_{M}\left(k\right) \end{array}$$

where $A_{P}=A_{M}-C_{M}D_{M}$, $B_{S}=B_{M}$, $C_{S}=D_{M}$. Similarly, by letting $a_{M}\left(k\right)=0$, *N(z)* in state space form is given as:

(7) $$\{\begin{array}{c} x_{M}\left(k+1\right)=A_{P}x_{M}\left(k\right)+B_{N}e_{M}\left(k\right)\\ l_{M}\left(k\right)=C_{N}x_{M}\left(k\right)+e_{M}\left(k\right) \end{array}$$

where $B_{N}=C_{M}$, $C_{N}=D_{M}$. Additionally, define E(k) as the unfiltered quantization noise, where

(8) $$E\left(k\right)=(l_{M}\left(k\right)-S\left(z\right)a_{M}\left(k\right))$$

Therefore,

(9) $$e_{M}\left(k\right)=N{\left(z\right)}^{-1}(l_{M}\left(k\right)-S\left(z\right)a_{M}\left(k\right))$$

The state space function for $N{\left(z\right)}^{-1}$ can be presented as:

(10) $$\{\begin{array}{c} x_{f}\left(k+1\right)=A_{F}x_{f}\left(k\right)+B_{F}E\left(k\right)\\ e_{M}\left(k\right)=C_{F}x_{f}\left(k\right)+E\left(k\right) \end{array}$$

where $A_{F}=A_{M}$, $B_{F}=C_{M}$, $C_{F}=-D_{M},$ and $x_{f}\in R$ is the state variable of $N{\left(z\right)}^{-1}$. 

#### 3.2.2. Unconstrained OBMPC Implementation

**Figure 7:**
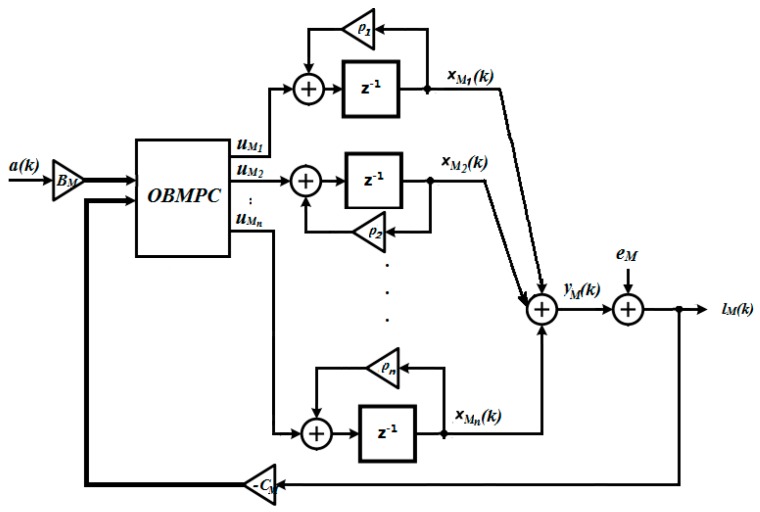
The OBMPC design for an nth order Δ∑ modulator (Thick lines denote vector routing).

The goal here is to implement a control structure that minimizes the filtered quantization noise $e_{M}\left(k\right)$. A conceptual view of an OBMPC-based Δ∑ modulator is shown in Figure 7:

In Figure 7, $u_{M}={\left[u_{M}{}_{1},u_{M}{}_{2}\ldots u_{M}{}_{n}\right]}^{T}$ is the control input that attempts to minimize the filtered quantization noise in Equation (11). An OBMPC is used in the modulation loop. The benefits of doing this are two-fold: Firstly, hard constraints can be easily included for each decoupled state variable, which provides more flexibility for the designer and the stability criteria can be easily acquired. Secondly, future predictions can be included in the control structure. Theoretically speaking, if the prediction horizon is long enough, then the quantization noise should tend towards zero. 

**Figure 8:**
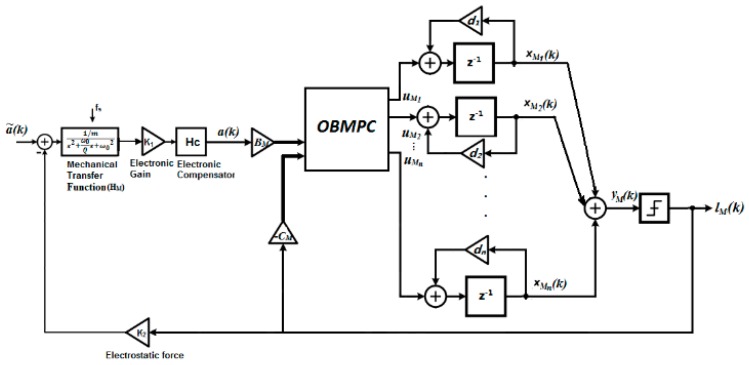
MEMS gyroscope using the OBMPCbased Δ∑ modulator (Thick lines denote vector routing).

Based on Figure 2 and Figure 7, a structure of the OBMPC based MEMS gyroscope can be designed as shown in Figure 8.

In Figure 8, $\tilde{a}\left(k\right)$ is the signal applied to the MEMS gyroscope, a(k) is the sensing signal picked up by the MEMS gyroscope which is the narrow band of interest around the gyroscope resonant frequency and $l_{M}\left(k\right)$ is the quantizer output under the OSR. The optimal solution $u_{M}{}^{*}\left(k\right)$ has an affine relationship between the quantized output and multi-bit coefficients and therefore forms a 1-bit processing structure. Specifically, define $H_{M}\left(z\right)$ and $H_{C}$(*z*) as the transfer function of the discretized gyroscope dynamic model and the compensator, respectively. For the OBMPC controller presented above, define the prediction horizon N and vectors:

$$\begin{array}{c} {\vec{e}}_{M}\left(k\right)≜{\left[e\left(k|k\right)e\left(k+1|k\right)\ldots e\left(k+N|k\right)\right]}^{T};\\ {\vec{u}}_{M}\left(k\right)≜{\left[u_{M}\left(k|k\right)u_{M}\left(k+1|k\right)\ldots u_{M}\left(k+N-1|k\right)\right]}^{T};\\ \vec{E}\left(k\right)≜{\left[E\left(k|k\right)\;E\left(k+1|k\right)\ldots E\left(k+N-1|k\right)\right]}^{T}. \end{array}$$

According to Equations (9) and (10):

(11) $$\{\begin{array}{c} {\vec{e}}_{M}\left(k\right)=F_{M}x_{M}\left(k\right)+\vartheta_{M}\vec{E}\left(k\right)\\ \vec{E}\left(k\right)={\vec{l}}_{M}\left(k\right)-S\left(z\right)\vec{a}\left(k\right) \end{array}$$

where $F_{M}=[\begin{array}{c} C_{F}\\ C_{F}A_{F}\\ C_{F}A_{F}{}^{2}\\ \vdots \\ C_{F}A_{F}{}^{N} \end{array}];{𝜗}_{M}=[\begin{array}{ccccc} D_{F} & 0 & 0 & \cdots & 0\\ C_{F}B_{F} & D_{F} & 0 & \cdots & 0\\ C_{F}A_{F}B_{F} & C_{F}B_{F} & D_{F} & \cdots & 0\\ \vdots & \vdots & \vdots & \ddots & \vdots \\ C_{F}A_{F}{}^{N-1}B_{F} & C_{F}A_{F}{}^{N-2}B_{F} & CA_{F}{}^{N-3}B_{F} & \cdots & D_{F} \end{array}]$.

${\vec{l}}_{M}\in R^{N}$ and $\overset{\text{⇀}}{a}\left(k\right)\in R^{N}$ are the predicted modulator input and output, respectively, along the prediction horizon. The cost function of Equation (11) can be built to minimize the filtered quantization noise:

(12) $$J\left(x_{M},{\vec{u}}_{M}\left(k\right)\right)=\underset{{\vec{u}}_{M}\left(k\right)}{\min}\left\{{\left|\left|x_{f}\left(k+N|k\right)\right|\right|}_{P_{M}}^{2}+\overset{N-1}{\underset{i=0}{\sum }}\left({\left|\left|x_{f}\left(k+i|k\right)\right|\right|}_{Q_{M}}^{2}+{\left|\left|u_{M}\left(k+i\right)\right|\right|}_{R_{M}}^{2}\right)\right\}$$

where $P_{M},Q_{M}$ and $R_{M}$ are positively defined weighting matrices. Define the main loop filter $L\left(z\right)≜{\left(zI-A_{M}\right)}^{-1}$ and a control input $u_{M}={\left[u_{M}{}_{1},u_{M}{}_{2}\ldots u_{M}{}_{n}\right]}^{T}$ as shown in Figure 8. Under the context of the MPC algorithm, if no constraint is applied, the MPC gain can be obtained by solving Equation (12) as:

(13) $$K_{M}=-\Phi^{-1}\Omega$$

where $\Omega ≜\Sigma_{i=1}^{N_{p}}(\sum_{j=0}^{i-1}A_{F}{}^{i-j-1}B_{F})Q_{M}A_{F}{}^{i}\;\in R^{N_{p}\times n}$, and $\Phi ≜\Sigma_{i=1}^{N_{p}}(\sum_{j=0}^{i-1}A_{F}{}^{i-j-1}B_{F})Q_{M}{\left(\sum_{j=0}^{i-1}A_{F}{}^{i-j-1}B_{F}\right)}^{T}+R_{M}\in R^{N_{p}\times N_{p}}$, where $Q_{M}≜\mathrm{diag}\left(Q_{M},Q_{M}\ldots .P_{M}\right)\in R^{N_{p}n\times N_{p}n},\;\mathrm{and}\;R_{M}=\mathrm{diag}\left(R_{M},R_{M}\ldots .R_{M}\right)\in R^{N_{p}\times N_{p}}$.

The global optimal solution of Equation (12) can be found as:

(14) $${\vec{E}}^{*}\left(k\right)=-K_{M}x_{f}\left(k\right)$$

Based on Equation (11), the state variable of $N{\left(z\right)}^{-1}$ can be derived as:

(15) $$x_{f}\left(k\right)={\left(zI-A_{F}\right)}^{-1}B_{F}\left(S\left(z\right)a\left(k\right)-l_{M}\left(k\right)\right)$$

By substituting Equation (15) into Equation (14), Equation (16) is obtained:

(16) $${\vec{E}}^{*}\left(k\right)=-K_{M}{\left(zI-A_{F}\right)}^{-1}B_{F}\left(S\left(z\right)a\left(k\right)-l_{M}\left(k\right)\right))$$

By substituting Equation (11) into Equation (16) and applying the receding horizon principle, the optimal modulator output $l^{*}\left(k\right)$ can be found as:

(17) $$l_{M}{}^{*}\left(k\right)=q\left(\left[1,0\ldots .,0\right]\left(S\left(z\right)a\left(k\right)+K_{M}{\left(zI-A_{F}\right)}^{-1}B_{F}\left(S\left(z\right)a\left(k\right)-l_{M}\left(k\right)\right)\right)\right)$$

Let the quantizer input be:

(18) $$y_{M}\left(k\right)=\left[1,0\ldots .,0\right]\left(S\left(z\right)a\left(k\right)+K_{M}{\left(zI-A_{F}\right)}^{-1}B_{F}\left(S\left(z\right)a\left(k\right)-l_{M}\left(k\right)\right)\right)$$

According to Equation (18), given the main loop filter L(z), the control input $u_{M}\left(k\right)$ can be determined as:

(19) $$u_{M}{}^{*}\left(k\right)=\left[1,0\ldots .,0\right]L{\left(z\right)}^{-1}(\left[1,z\ldots z^{n-1}\right]S\left(z\right)a\left(k\right)+K_{M}{\left(zI-A_{F}\right)}^{-1}B_{F}\left(S\left(z\right)a\left(k\right)-l_{M}\left(k\right)\right)$$

By reformatting Equation (19), we obtain:

(20) $$u_{M}{}^{*}\left(k\right)=\left[1,0\ldots .,0\right]L{\left(z\right)}^{-1}\left(\left(\left[1,z\ldots z^{n-1}\right]+K_{M}{\left(zI-A_{F}\right)}^{-1}B_{F}\right)S\left(z\right)a\left(k\right)-K_{M}{\left(zI-A_{F}\right)}^{-1}B_{F}l_{M}\left(k\right)\right)$$

By assigning $L_{0}=L{\left(z\right)}^{-1}\left(\left(\left[1,z\ldots z^{n-1}\right]+K_{M}{\left(zI-A_{F}\right)}^{-1}B_{F}\right)S\left(z\right)\right)$, and $L_{1}=L{\left(z\right)}^{-1}K_{M}{\left(zI-A_{F}\right)}^{-1}B_{F}$, Equation (20) can be re-written as:

(21) $$u_{M}{}^{*}\left(k\right)=\left[1,0\ldots .,0\right]\left({\vec{L}}_{0}a\left(k\right)-{\vec{L}}_{1}l_{M}\left(k\right)\right)$$

Furthermore define $H_{A}\left(z\right)≜K_{1}H_{M}\left(z\right)\;H_{C}\left(z\right)$, then according to Figure 8

(22) $$a\left(k\right)≜H_{A}\left(z\right)\left(\tilde{a}\left(k\right)-K_{2}l_{M}\left(k\right)\right)$$

By substituting Equation (22) into Equation (21), the optimal control input can be obtained as:

(23) $$u_{M}{}^{*}\left(k\right)=\left[1,0\ldots .,0\right]\left(L_{0}H_{A}\left(z\right)\tilde{a}\left(k\right)-\left(L_{1}+L_{0}H_{A}\left(z\right)K_{2}\right)l_{M}\left(k\right)\right)$$

where $L\_0=L{\left(z\right)}^{-1}(\left(\left[1,z\ldots z^{n-1}\right]+K_{M}{\left(zI-A_{F}\right)}^{-1}B_{F}\right)S\left(z\right)$, and $L_{1}=L{\left(z\right)}^{-1}K_{M}{\left(zI-A_{F}\right)}^{-1}B_{F}$.

Note that the gyroscope output $\tilde{a}$ relatively slow comparing to the oversampled modulator output. Hence, $\tilde{a}$ can be regarded as a constant signal within l time steps (l = OSR), i.e., $\tilde{a}\left(k\right)=\tilde{a}\left(k+1\right)\ldots =\tilde{a}\left(k+l\right)$ and the first component in Equation (23), i.e., $L_{0}H_{A}\left(z\right)\tilde{a}\left(k\right)$, can be calculated under a relatively low sampling rate. If the quantization levels are standardized into ±1, i.e., $l_{M}\left(k\right)$ = ±1, it can be seen from Equation (23) that the optimization is based on the gyroscope output $\tilde{a}\left(k\right)$ and the bi-level modulator output. Hence, for the second component in Equation (23), i.e., $\left(L_{1}+L_{0}H_{A}\left(z\right)K_{2}\right)l_{M}\left(k\right)$, the multiplications in the proposed structure are between a 1-bit signal and a multi-bit controller coefficient. This operation, in fact, just changes the sign of the multi-bit coefficient, which removes the multiplier from the controller, forming a multiplier free structure.

#### 3.2.3. Hard Constraints on OBMPC based MEMS Gyroscope

The design of the Δ∑ modulator with parallel state variables provides more flexibility in terms of designing the constraints of each state variable. The constraint levels can be determined by studying the impulse response of each integrator or simply according to Equation (3). In the proposed parallel state structure, even if the state variable cannot be fully decoupled, the structure in each branch will still be relatively simple (typically second order if the coupling is merely caused by the resonator). Hence, it is relatively easy to determine a reasonable constraint level to each state variable.

Given a set of constraints applied to the state variables:

(24) $$G_{M}x_{M}\left(k\right)\leq \gamma_{M}$$

where $G_{M}$ and $\gamma_{M}$ are acquired based on the state bound for each integrator which can guarantee the stability of the modulator. The Hildreth’s QP procedure [42] is used in this work as an iteration method for the MPC algorithm. The same method has been adopted in [43] as an iteration method for the MPC algorithm. 

When a set of active constraints is applied to the quantizer input *a(k)*, the optimal solution can be solved iteratively by introducing the modified Lagrange factor $\bar{\lambda }$:

(25) $$u_{M}{}^{*}\left(k\right)=L_{0}\tilde{a}\left(k\right)-L_{1}l_{M}\left(k\right)-\bar{\lambda }$$

where $\bar{\lambda }\in$ *R_p_* and ${\bar{\lambda }}_{i}={\bar{S}}_{i}+{\bar{W}}_{\left(i,:\right)}{\left(zI-A_{F}\right)}^{-1}B_{F}\left(S\left(z\right)a\left(k\right)-l_{M}\left(k\right)\right))$. $\bar{S}\;\mathrm{and}\;\bar{W}$ are the corresponding matrices are solved during the dual level iteration process. Hildreth’s QP algorithm is based on an element-by-element search and it does not require any matrix inversion. Therefore, the program will continue without interruption even when the rows of G are not linearly independent (e.g., more than one constraints are active). Also, $\bar{\lambda }$ is a near-optimal solution in a finite iteration loop. The iteration expression of Hildreth’s QP procedure is given in the following equation:

(26) $${\bar{\lambda }}_{i}^{m+1}=max\left(0,\omega_{i}^{m+1}\right)$$

where $\omega_{i}^{m+1}=-\frac{1}{h_{ii}}\left[k_{i}+\sum_{j=1}^{i-1}h_{ij}{\bar{\lambda }}_{j}^{m+1}+\sum_{j=i+1}^{n}h_{ij}{\bar{\lambda }}_{j}^{m}\right]$, and *m* means the *m*th iteration, the scalar $h_{ii}$ is the *ii*th element in the matrix $H=G_{M}\Phi^{-1}G_{M}{}^{T}$ and $k_{i}$ is the *i*th element in the vector $K=\gamma_{M}+G_{M}G\Phi^{-1}\Omega \tilde{x}\left(k\right)$. $\bar{\lambda }\left(k\right)$ is calculated according to the previous one, $\bar{\lambda }\left(k-1\right)$, which can either be 0 or an affine function of the quantized measurement $\tilde{x}\left(k-1\right)$. Given a finite number of iteration, $\bar{\lambda }$ can be solved as a near optimal solution even if two or more constraints are active at the same time. Therefore, even if $\bar{\lambda }\left(k\right)$ cannot be solved explicitly, a set of near optimal control input U(k) can still be found as an affine function over the state feedback $\tilde{x}\left(k\right)$.

If one or several of the constraints are violated, then ${\bar{\lambda }}_{i}$ will be calculated accordingly. Note that since both ${\bar{\lambda }}_{i}$ and the global optimal solution presented in Equation (24) have an affine relationship with the 1-bit feedback $l_{M}\left(k\right)$ (once again a(k) will be considered as constant within several time steps), the arithmetic block of the proposed OBMPC controller can process all the fast sampled operations with simple conditional-negates (CN) and bit shifters, and therefore achieves the 1-bit processing structure. In the proposed parallel structure, since the state variables are linearly independent, a simple active set method can be used to efficiently find the optimal *K_M_* and then in turn, find *H_a_* and *H_l_*.

## 4. Direct Implementation Method Using the OBMPC Approach

In the proposed structure, the hard constraints can be directly included into each of decoupled state variables according to the impulse response of each integrator. This can greatly simplify the design process. It is worth noting that Equation (13) is not strictly compliant with the 1-bit processing structure since $\tilde{a}\left(k\right)$ is the multi-bit counterpart of the analog signal. Modulating $\tilde{a}\left(k\right)$ into a 1-bit signal is not appropriate as this may increase the circuit complexity and introduce additional quantization noise into the control loop. However, as the system is oversampled, $\tilde{a}\left(k\right)$ is relatively slow in comparison to the sampling rate. Hence, the computation burden is mainly caused by the second component in Equation (13), i.e., $\left(L_{1}+L_{0}H_{A}\left(z\right)K_{2}\right)l_{M}\left(k\right)$. If the bi-level quantizer is adopted, then the explicit relationship between the multi-bit parameters and the bi-level quantized signal can provide a multiplier free structure. Hence, the circuit simplicity of the 1-bit Δ∑ modulator will be preserved. 

**Figure 9:**
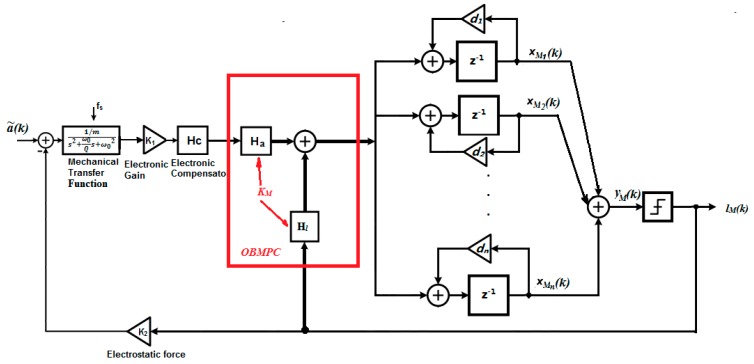
Design of a high order1-bit MEMS gyroscopeusing the OBMPC.

Essentially, the proposed OBMPC changes the zeros of the NTF by finding the optimal solution that minimizes the filtered quantization noise and therefore improves the performance of the system. For applications where the Δ∑ modulator is to be constructed using analog components (i.e., controllers are not feasible in the modulation loop), the MPC approach can still be treated as a design guideline to design a higher order Δ∑ modulator. The $K_{M}$ that is solved by the MPC can change the zeros of the NTF and therefore affect the noise shaping characteristics of the modulator. According to Equation (23), define $H_{a}\left(z\right)={\left[\frac{L_{0}{}_{1}}{b_{1}},\frac{L_{0}{}_{2}}{b_{2}}\ldots \frac{L_{0}{}_{n}}{b_{n}}\right]}^{T}$ and $H_{l}\left(z\right)={\left[-\frac{L_{1}{}_{1}}{c_{1}},-\frac{L_{1}{}_{2}}{c_{2}}\ldots -\frac{L_{1}{}_{n}}{c_{n}}\right]}^{T}$, then Figure 8 can be simplified as Figure 9. 

The $K_{M}$ in Figure 9 can be regarded as the functional scaling factor on each integrator to achieve a certain ${\vec{L}}_{0}$ and ${\vec{L}}_{1}$. By designing the appropriate values for $P_{M}$ and $Q_{M}$ (i.e., to satisfy $A_{M}{}^{T}P_{M}A_{M}+Q_{M}=P_{M}$), the modulator can be safely scaled while the stability of the control loop is guaranteed. However, a major disadvantage of this approach is that the constraints cannot be directly included which means clipping or a different stabilization technique needs to once again be used in the modulator.

## 5. Numerical Example

In the interest of justifying the OBMPC controller in the MEMS gyroscope design, the simulation in this section focuses on the sense mode of the gyroscope. The input signal is acted upon by the proof mass of a second order spring and damping mechanical system as stated in Equation (1). The proof mass of the sense mode is $m=1.96\times 10^{-9}$ kg. The quality factor is set as $Q_{f}=100$ and the resonance frequency of the mechanical system is 4000Hz. The quantization level is standardized into ±1 V, and translated into the electrostatic feedback force by the gain of the voltage to force conversion $K_{2}=3.35\times 10^{-9}\;V/N$. The input signal is first defined as a periodic input signal operating at 64 rad/s with an amplitude of 0.6 rad/s.

**Figure 10:**
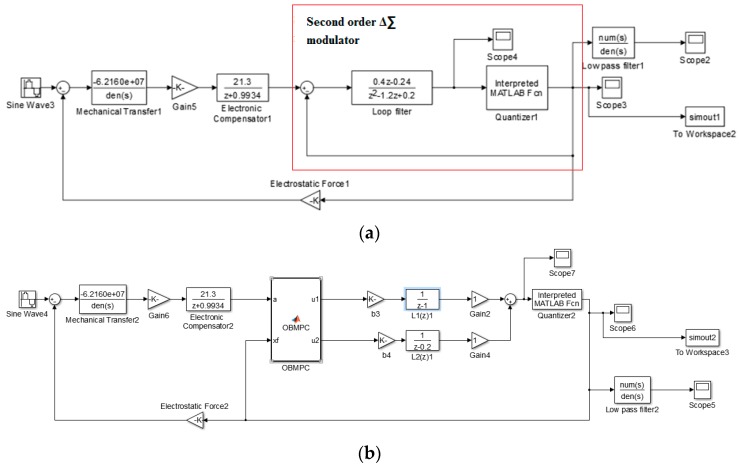
(**a**) Simulation structure of the Δ∑ modulator based MEMS gyroscope; (**b**) Simulation structure of the OBMPC-based MEMS gyroscope.

The structure of the MEMS gyroscope is shown in Figure 10a. The sampling time is set to $1.625\times 10^{-9}$ s (OSR = 200). A lead compensator is used to deal with the phase shift introduced by the mechanical sensing element. A simple second order Δ∑ modulator is presented here as $A_{M}=\left[\begin{array}{cc} 1 & 0\\ 0 & 0.2 \end{array}\right]$, $B_{M}=\left[\begin{array}{c} 0.2\\ 0.2 \end{array}\right]$, $C_{M}=\left[\begin{array}{c} 0.2\\ 0.2 \end{array}\right],\;D_{M}=\left[\begin{array}{cc} 1 & 1 \end{array}\right]$. The state space realization is shown in Equation (27):

(27) $$\{\begin{array}{c} x_{M}\left(k+1\right)=\left[\begin{array}{cc} 1 & 0\\ 0 & 0.2 \end{array}\right]x_{M}\left(k\right)+\left[\begin{array}{c} 0.2\\ 0.2 \end{array}\right]a_{M}\left(k\right)-\left[\begin{array}{c} 0.2\\ 0.2 \end{array}\right]l_{M}\left(k\right)\\ y_{M}\left(k\right)=\left[\begin{array}{cc} 1 & 1 \end{array}\right]\left[\begin{array}{c} x_{M}{}_{1}\\ x_{M}{}_{2} \end{array}\right] \end{array}$$

The filter $N{\left(z\right)}^{-1}$ can then be denoted as:

(28) $$\{\begin{array}{c} x_{f}\left(k+1\right)=\left[\begin{array}{cc} 1 & 0\\ 0 & 0.2 \end{array}\right]x_{f}\left(k\right)-\left[\begin{array}{c} 0.2\\ 0.2 \end{array}\right]E\left(k\right)\\ e\left(k\right)=\left[\begin{array}{cc} -1 & -1 \end{array}\right]x_{f}\left(k\right)+\;E\left(k\right) \end{array}$$

If no constraint is applied and *N =* 4, then $K_{M}$ can be found as

(29) $$K_{M}=\left[\begin{array}{cc} -0.1584 & -0.0240\\ -0.1411 & -0.0040\\ -0.1245 & -0.0002\\ -0.1064 & -0.0005 \end{array}\right]$$

Consequently, ${\vec{L}}_{0}$ and ${\vec{L}}_{1}$ can be found as:

(30) $${\vec{L}}_{0}=\frac{z^{2}-1.2365z+0.2111}{z^{2}-0.8z-0.04}$$

(31) $${\vec{L}}_{1}=\frac{0.0365z-0.0111}{0.4z-0.24}$$

Based on the above equations, the control structure is shown in Figure 10b in comparison of a second order Δ∑ modulator structure.

We first assume the system is ideal (e.g., no electrical noises and internal distortion exist). Then the angular system input is a sinusoidal input. The delay caused by the filter is compensated in the generated result. A second order Δ∑ modulator based gyroscope is designed for benchmarking purposes.

**Figure 11:**
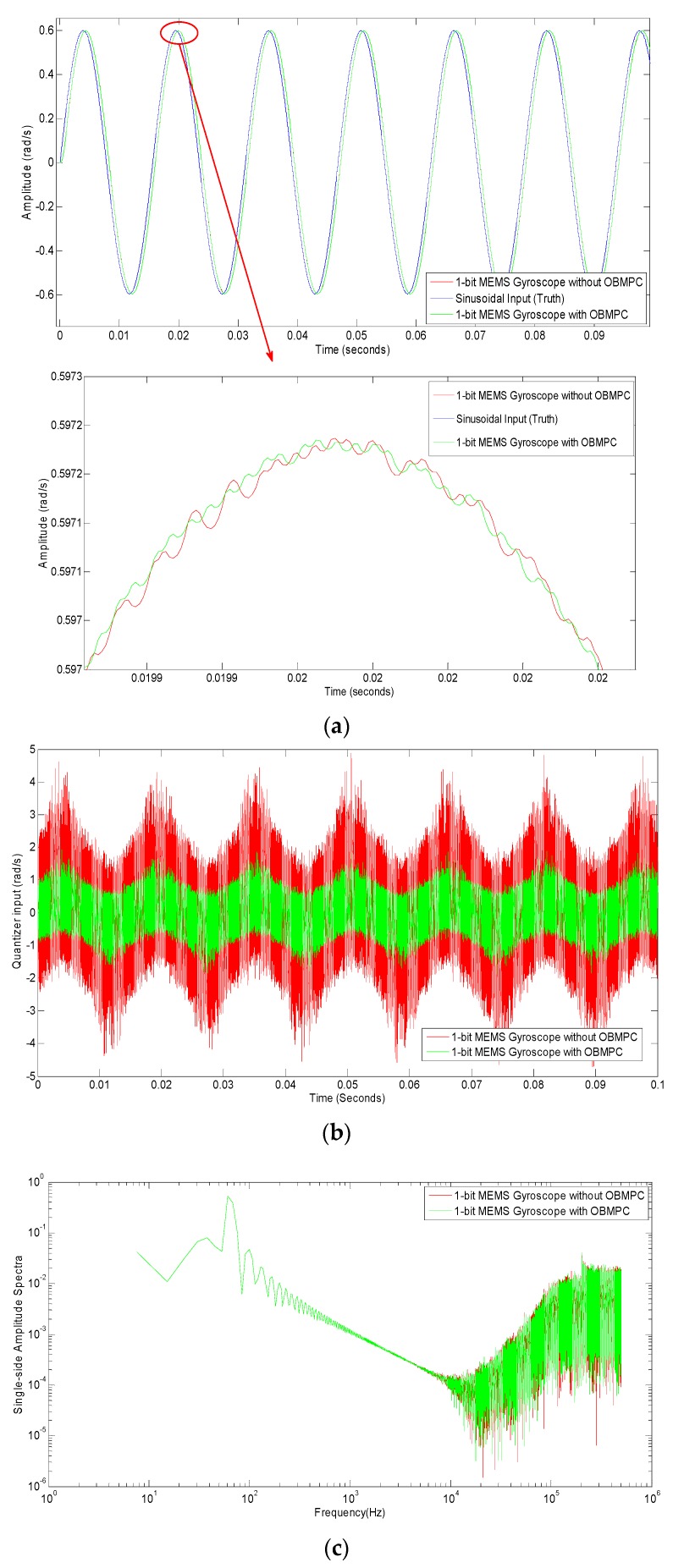
Results for the Δ∑ modulator-based MEMS gyroscopes with Amplitude = 0.6 rad/s: (**a**) MEMS gyroscopes with sinusoidal input; (**b**) Comparison of the quantizer input; (**c**) Spectra comparison.

The tracking trajectory and the spectra of both systems are plotted in Figure 11. As shown in Figure 11a,c, both systems show good tracking results to the input signal. The amplitude of the quantizer input shows some difference but none of them reached the constraint (as shown in Figure 11b). It can be seen from Figure 11c that the spectra are not much different (as expected) since no constraints are applied to the system. However, the OBMPC tends to be better than its benchmark when higher frequency is applied. This is due to the fact that the low pass filter in the designed system is less sufficient in the benchmark than the one in the OBMPC structure. This point will be further verified in the following simulations. 

**Figure 12:**
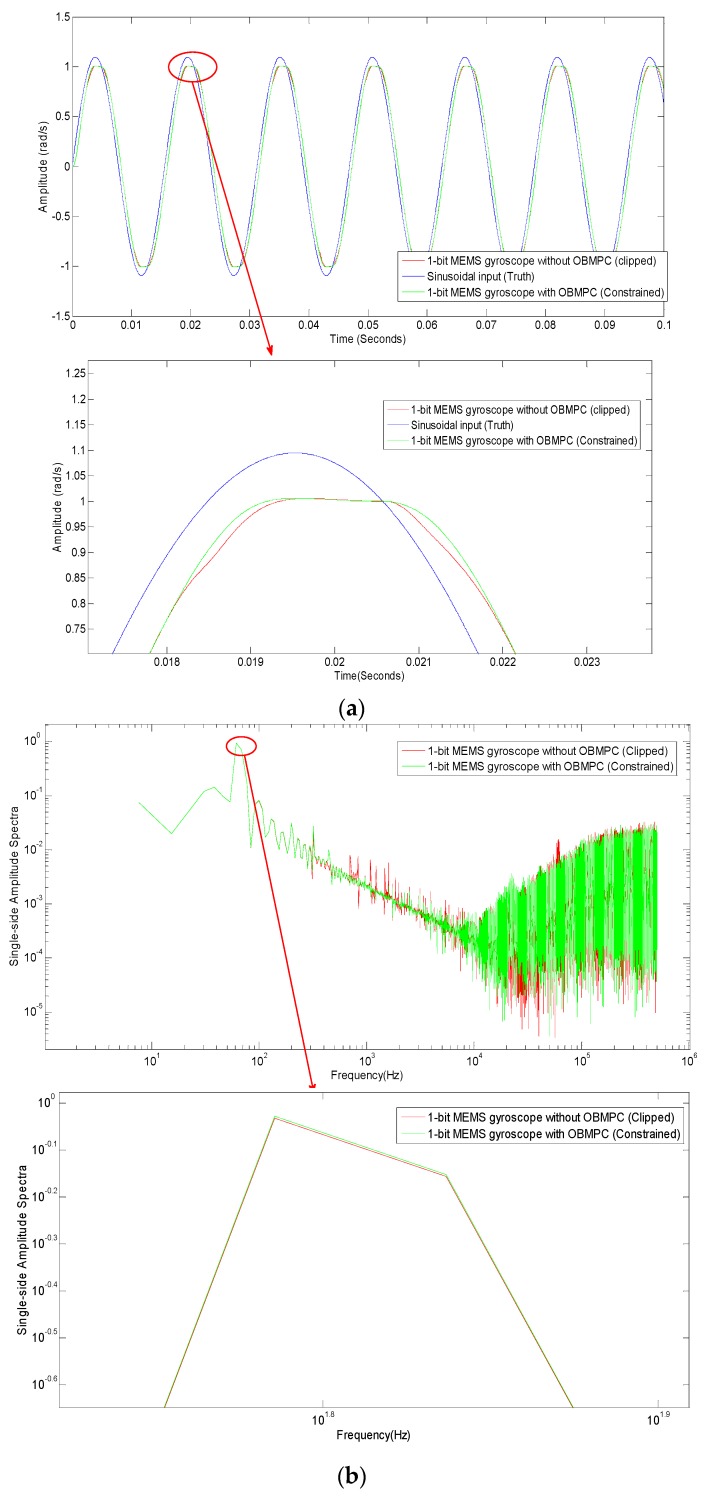

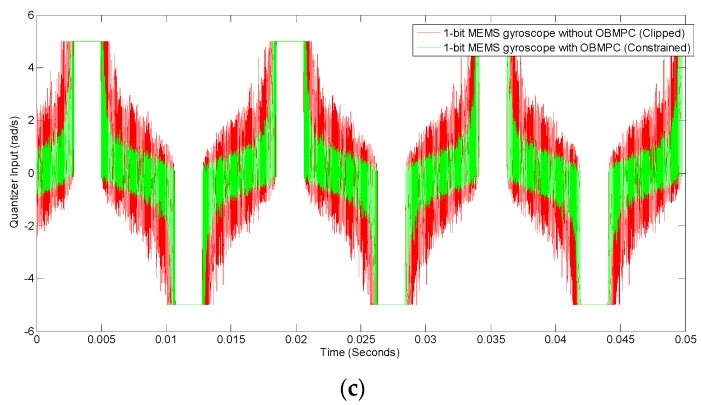
Results for the OBMPC-based MEMS gyroscope with Amplitude = 1.1 rad/s (**a**) MEMS gyroscope with sinusoidal input; (**b**) Spectra comparison; (**c**) Comparison of the quantizer input.

The amplitude of the input signal shall now be increased to 1.1 rad/s, so that the quantizer will be overloaded. Constraints and clippers are set to both systems respectively according to Equation (2). The tracking trajectory and the spectra of both systems are plotted in Figure 12. In this scenario, the OBMPC-based MEMS gyroscope shows a notable improvement in comparison to its benchmark. The trajectory of the amplitude is closer to the input signal around the overloading area (as shown in Figure 12a,c) and the quantizer input is nicely shaped (as shown in Figure 12b) as the hard constraints are handled better by the OBMPC than the clipper. The spectra also show that the OBMPC based MEMS gyroscope performs better at both the peak (as shown in Figure 12c) and at higher input frequency (as shown in Figure 12b). The OBMPC based Δ∑ modulator shows less noise leakage at the higher frequency as the OBMPC controller improves the original low pass filter in the loop. 

**Figure 13:**
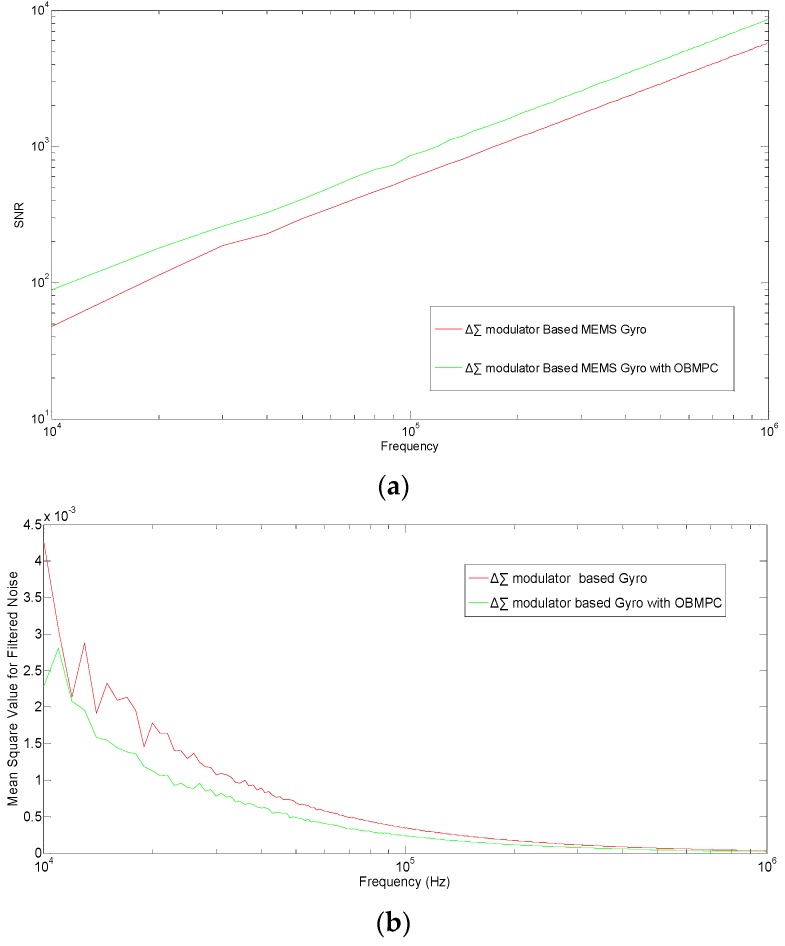
(**a**) SNR and (**b**) MSE of the quantization noise with different sampling frequency.

To further discuss the character of the proposed MEMS gyroscope, the OBMPC-based MEMS is compared with its benchmark under different sampling frequencies. The SNR and the MSE of the quantization noise are plotted in Figure 13.

**Figure 14:**
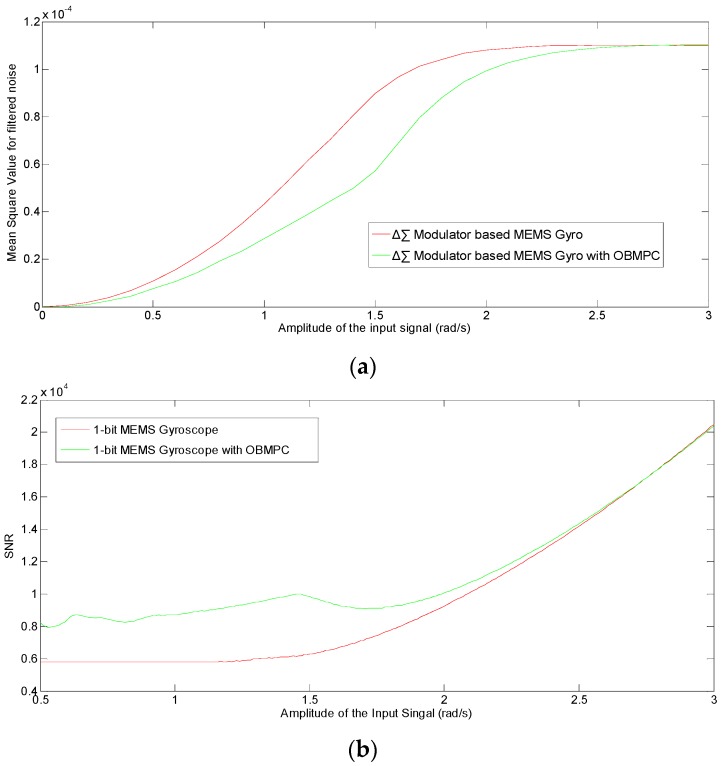
(**a**) MSE and (**b**) SNR of the quantization noise with different input amplitude.

It can be seen that under high sampling frequencies (>10^4^ Hz), the OBMPC-based MEMS gyroscope provide better SNR and lower noise level than its benchmark due to the use of MPC controller. Moreover, since the amplitude of the modulator input signal is important to the performance of the Δ∑ modulator based system, the SNR and MSE of the quantization noise under different input signal amplitudes are obtained as shown in Figure 14.

Figure 14 once again shows that the OBMPC based MEMS gyroscope provides better SNR and lower noise level than its benchmark due to the use of MPC controller. As the input amplitude approaches the quantization level, the quantization noise become even higher due to the integrators in the loop and may trigger the clippers or constraints in the respective design circuits. In this case, the clipper only chops the amplitude of the quantizer input rather than calculating the optimal value towards the cost function of the noise transfer function. Hence, the OBMPC-based MEMS gyroscope provides better SNR over the conventional Δ∑ modulator-based MEMS gyroscope. It is worth noting that if the amplitude of the input signal is much higher than the quantization level, then the system will no longer track the input signal and both systems will need to be redesigned (e.g., change the quantization level or the loop gain). 

**Figure 15:**
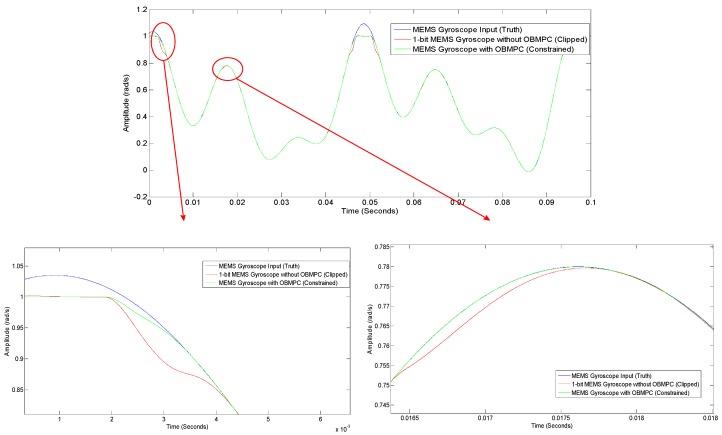
Comparisons between the OBMPC gyroscope and the conventional Δ∑ modulator-based gyroscope.

Finally, a random input with a random noise of relatively high amplitude and low frequency added to each of the integrators shall be simulated. The response of the Δ∑ modulator-based MEMS gyroscope for this scenario is given in Figure 15. It can be seen that the OBMPC-based MEMS gyroscope tracks the input signal better than its benchmark, especially around the quantization level, which proves the benefits of using the OBMPC controller in the MEMS gyroscope.

## 6. Conclusions

In this paper, an OBMPC algorithm for the 1-bit MEMS gyroscope was introduced. The challenges of the Δ∑ modulator-based MEMS gyroscope are mainly the randomness of the sensor input and the noise introduced by the mechanical and electrical systems. Hence, it is essential to improve the SNR while maintaining the robustness of the control system of the gyroscope control loop. In this work, we propose to include an OBMPC algorithm in the Δ∑ modulator-based gyroscope. Such a structure improves the SNR by minimizing the filtered quantization noise. As a result, constraints applied on the modulator state variables can be included in the controller directly, and an optimized gain can be determined so that the coefficients can be safely scaled. Due to the 1-bit nature of the modulated signal, if the state variable can be decoupled, so that the constraints applied to the state variable are linearly independent, the OBMPC can be implemented with relatively simple circuit under the framework of the 1-bit processing control system. Note that it is not necessary to apply constraints to all the state variables to stabilize the sensing output. Hence, if some of the modulator states cannot be decoupled, the constraint can be applied to the last state variable in that branch. 

Simulation results show that the OBMPC-based MEMS gyroscope with a 2nd-order transfer function can provide better SNR than its conventional counterpart, especially when the signal amplitude is near the constrained levels. Also, the constraints of the MEMS gyroscopes can be designed to achieve guaranteed stability. For a higher order MEMS gyroscope model, the whole feedback loop with the OBMPC controller, the dynamic model and the delta sigma modulator can be regarded as a higher order delta sigma modulator. In this case, this will increase the system’s noise shaping capability and subsequently improve the signal-to-noise ratio.
